# Influence of Malnutrition on Clinical Outcomes in Patients With Hip Fractures in a Trauma Unit: An Observational Study

**DOI:** 10.7759/cureus.90286

**Published:** 2025-08-17

**Authors:** Li Cheng Chong, Alia Shaaban, Oday Al-Dadah

**Affiliations:** 1 Department of Trauma and Orthopaedic Surgery, South Tyneside District Hospital, South Shields, GBR; 2 Translational and Clinical Research Institute, Faculty of Medical Sciences, Newcastle University, Newcastle upon Tyne, GBR

**Keywords:** albumin, hip fracture, malnutrition, mortality, nutrition status, surgery

## Abstract

Introduction: Hip fractures are most prevalent in the elderly population, who are also at high risk of malnutrition. Malnutrition is a significant risk factor for poor functional recovery and a higher risk of mortality. This study aimed to evaluate the influence of nutrition status on clinical outcomes in patients with hip fractures.

Methods: An observational study of all patients with hip fractures in the year 2020 admitted to South Tyneside District Hospital, a district general hospital trauma unit in South Shields, England, was conducted. Data collected included patient demographics, nutrition status (Malnutrition Universal Screening Tool (MUST)), body mass index (BMI), mortality, biochemical markers, delirium score, acute kidney injury (AKI) status, time to surgery, and length of hospital stay.

Results: A total of 188 patients were included in this study. Normal nutrition was observed in 137 patients (73%), whilst 51 patients (27%) were categorized as at risk of malnutrition. The latter group comprised older patients (p=0.005). Patients at risk of malnutrition demonstrated an increased rate of mortality (p<0.001), with mortality rates at 63% as compared to 30% for patients with normal nutrition. They were also associated with increased risk for the development of delirium (p=0.001) and length of hospital stay (p=0.034) as compared to patients with normal nutrition. There was a significant between-group difference of serum albumin, CRP, estimated glomerular filtration rate (eGFR), and urea (p<0.05).

Conclusion: The majority of patients with hip fractures were elderly females with multiple comorbidities. Approximately one-quarter of patients were deemed at risk of malnutrition. Advanced age significantly contributed to developing malnutrition, which in turn increased the rate of mortality, delirium, and length of hospital stay. Early nutritional assessment and intervention are paramount in reducing the associated risks and enhancing clinical outcomes.

## Introduction

Hip fractures represent a major public health concern, with annual incidence in the UK estimated to be around 76,000 cases per year, predominantly affecting the elderly population aged above 70 years [1]. This number is projected to increase, with global prevalence estimated to reach a total of 2.6 million by 2025 [2]. Hip fractures are significant injuries that not only have an immediate impact on patients, including pain, loss of mobility, prolonged recumbency, and pressure sores, but can also profoundly impact an individual’s health and well-being in the long term, resulting in functional decline, loss of independence, and increased risk of mortality [3]. As such, it is important to prevent fragility fractures where possible and to optimize the care of patients with hip fractures to achieve a favorable clinical outcome.

The mainstay management of hip fractures, if medically fit for general anesthesia, is surgical intervention [4]. The recovery from a hip fracture can be compounded by non-modifiable factors such as age, gender, and comorbidities [5]. However, modifiable factors that could optimize recovery include adequate nutrition, as detailed in fragility fracture guidelines from the British Orthopaedic Association, which emphasizes its importance [6]. However, in clinical practice, the focus in the recovery period tends to be on physiotherapy, thromboprophylaxis, and secondary prevention for bone health, with patients’ current nutritional status often being overlooked [1]. Several studies have shown that the nutritional intake is often inadequate in patients admitted to hospital with hip fractures, particularly in the older cohort, with the majority frequently not meeting the energy and protein requirements as compared to age-matched patients without hip fractures [6-8].

The utilization of nutrition screening tools such as the Malnutrition Universal Screening Tool (MUST) can be a quick and effective way to identify patients at risk of malnutrition [9] and to optimize their nutritional intake. MUST is a five-step screening tool developed by the British Association for Parenteral and Enteral Nutrition (BAPEN) to identify adults at risk of malnutrition. It incorporates three key components, which are body mass index (BMI), unintentional weight loss, and the effect of acute disease on nutritional intake. Each component is scored and categorizes patients according to the risk of malnutrition.

The nutrition status of patients in the hospital is a critical aspect of their overall health and recovery [10]. Malnutrition is a common yet often under-recognized factor contributing to poor clinical outcomes and is a significant risk factor for poor functional recovery and longer hospital stays following hip fracture [10-11]. Recent studies have demonstrated that nutritional status significantly influences mortality, postoperative complications, and recovery trajectories in this population [12-14]. However, there remains a gap in understanding how early identification and management of nutritional risk can affect key biochemical markers and length of hospital stay in real-world hospital settings. This study aims to address this gap by evaluating the impact of nutritional status on clinical outcomes in patients with hip fractures, focusing specifically on mortality rate, postoperative complications, length of hospital stay, and key biochemical markers, thereby providing evidence to inform improved nutritional assessment and intervention strategies for patients with hip fractures.

This article was previously presented as a poster at the 2024 Association of Physicians of Great Britain & Ireland Annual Meeting on May 23-24, 2024.

## Materials and methods

This was a retrospective observational study conducted at South Tyneside District Hospital, a district general hospital trauma unit in South Shields, England. This study was exempt from Institutional Review Board (IRB)/Ethics Committee approval, as it was a pragmatic study evaluating the existing clinical practice of the host institution’s trauma and orthopaedic department. The hospital electronic health record system (MediTech version 6 (Medical Information Technology Inc., Westwood, Massachusetts, USA)) was used to collect data for all patients.

The inclusion criteria consisted of all patients admitted to the trauma unit of the district general hospital within the calendar year 2020, diagnosed with a hip fracture, and who had a nutritional assessment completed upon admission. The exclusion criteria were patients who did not have a nutritional assessment completed upon admission.

In terms of sampling technique, a combination of purposive and convenience sampling was used because the study aimed to explore nutritional status in a specific patient population within a defined clinical setting. Purposive sampling was applied to ensure inclusion of patients meeting predefined inclusion criteria that aligned with the study objectives. Convenience sampling was used due to the retrospective nature of the study and reliance on patients admitted during the study period whose records were accessible and sufficiently complete for analysis. This approach was chosen to maximize feasibility while still targeting the relevant clinical group.

Data collected included demographics, patient nutrition status, type of hip fracture surgery, American Society of Anesthesiologists (ASA) grade, acute kidney injury (AKI) status, pre- and postoperative serum biochemical markers (albumin, creatinine, CRP, estimated glomerular filtration rate (eGFR), potassium, sodium, and urea), delirium score (4AT score which comprises four components, including arousal, attention, the abbreviated mental test - 4 (AMT-4), and acute change) [15-16], postoperative fluid balance, mortality rate, time to surgery, and length of hospital stay. These data were collected within the time period of the same hospital admission from the day of admission to the day of discharge, except for the mortality rate, which was followed up to one year postoperatively.

MUST [17] is a nutritional screening tool developed by the BAPEN that can be applied to all adult patients in any care setting to detect malnutrition and guide management. The rationale for selecting the MUST score in our study is due to its clinical applicability in acute care settings, due to its simplicity, rapidity, and reliance on routinely available clinical parameters such as BMI, recent weight loss, and acute disease effect. This makes it highly feasible for early nutritional risk screening in the acute trauma population. Secondly, the MUST score is validated across a broad range of adult patients, unlike tools such as Mini Nutritional Assessment (MNA), which are specifically designed for older patients, making it more generalizable to the cohort in our study. Finally, the MUST score is the standard nutritional screening tool used at our institution. As such, utilizing this tool ensured consistency with routine clinical workflows and enhanced the applicability of our findings. Table 1 presents the five steps it comprises, which ultimately provide the overall risk of malnutrition and corresponding recommendations for management [18]. For clarity, the term “normal nutrition” is used to describe patients assessed to be “low risk,” i.e., with a score of 0, and the term “at risk of nutrition” is used to describe patients assessed to be “medium risk” and “high risk,” i.e., with scores of one and two, respectively.

**Table 1:** Malnutrition Universal Assessment Tool (MUST) score calculator Source: [17]

Steps	Instructions	Score
1: BMI	Measure height and weight to calculate a BMI score, denoted according to BMI category.	0 for BMI > 20 kg/m^2^
		1 for BMI between 18.5 to 20 kg/m^2^
		2 for BMI < 18.5 kg/m^2^
2: Weight loss	Calculate the percentage of unplanned weight loss in the preceding three to six months.	0 for weight loss < 5%
		1 for weight loss between 5% to 10%
		2 for weight loss > 10%
3: Acute disease	Establish the presence of concurrent illness and the status of nutritional intake.	2 for if the patient is acutely ill and will likely have no nutritional intake for > five days.
4: Total score	Add scores from steps 1 to 3 to obtain the overall risk of malnutrition.	Minimum score = 0; Maximum score = 6.
5: Management	Manage the patient according to the total score calculated in step 4.	0: Low risk; routine clinical care recommended.
		1: Medium risk; recommendation for observation (e.g., monitoring dietary intake for adequacy).
		≥ 2: High risk; recommendation to treat (e.g., referral to a dietician).

Statistical analysis

All continuous data distributions were evaluated by plotting histograms with fitted curve lines, box plots, normal Q-Q plots, and the Kolmogorov-Smirnov test. All the serum blood test data (continuous variables) displayed a skewed distribution, and the appropriate non-parametric statistical tests were used in their analyses. The Mann-Whitney U test was used for the between-group statistical analyses of continuous data. The chi-square test for independence was used for the statistical analyses of categorical variables. The level of statistical significance was set at p<0.05. Statistical analysis was undertaken using IBM SPSS Statistics for Windows, version 26.0 (IBM Corp., Armonk, New York).

## Results

A total of 190 patients who satisfied our inclusion criteria were identified through purposive and convenience sampling. Two patients did not have a nutritional assessment and, as such, were excluded from the study. This resulted in a total of 188 patients being included in the study. Among the 188 patients, 185 patients underwent operative management, and three patients were managed conservatively.

Table 2 presents the patient demographics. The majority were female (n=153), elderly with a mean age of 81.9 years, and frail with an ASA grading of three or above. Most had sustained a left-sided (n=110), intra-capsular (n=128) fracture. The MUST score demonstrated that the majority (n=137) had normal nutrition. No patients reached the threshold of established malnutrition.

**Table 2:** Patient demographics and clinical characteristics SD: standard deviation; BMI: body mass index; ASA: American Society of Anesthesiologists Physical Status Classification; MUST: Malnutrition Universal Assessment Tool

Variable	All (N=188)
Age, mean (range)	82 (60-103)
Sex, no. (%)	
Female	153 (81%)
Male	35 (19%)
Height (m), mean (SD)	1.62 (0.10)
Weight (m), mean (SD)	62.3 (15.0)
BMI (kg/m2), mean (SD)	23.6 (4.8)
Laterality, no. (%)	
Right	78 (41%)
Left	110 (59%)
Fracture type, no. (%)	
Intracapsular	128 (68%)
Extracapsular	60 (32%)
ASA score, no. (%)	
1	3 (1.6%)
2	58 (30.5%)
3	111 (60.0%)
4	13 (7.0%)
MUST score, no. (%)	
Normal nutrition	137 (73%)
At risk of malnutrition	51 (27%)
Established malnutrition	0 (0%)

Table 3 presents data on the onset of AKI in all patients recovering from hip fractures, including those managed conservatively or surgically. As AKI is a notable risk factor of mortality and often associated with increased risk of complications, it was clinically relevant to include this data in this study. The data of the patient group at risk of malnutrition were compared with the patient group with normal nutrition. However, there was no between-group difference found (p=0.733). In terms of vital status, both in-hospital and one-year mortality rates were analyzed, showing significantly higher mortality rates in the at-risk-of-malnutrition group as compared to the normal nutrition group (p<0.001). Using the formula (Patients who died / (Patients who lived + died) x 100) for each group, the mortality for the patients with normal nutrition was 30% (41/137 x 100), and that for patients at risk of malnutrition was 63% (32/51 x 100) for the entire cohort of 188 patients.

**Table 3:** Effect of nutrition on development of AKI and on mortality AKI: acute kidney injury; ^1^chi-square test for independence; X^2^ Pearson's chi-square; *statistically significant <0.05

Variable	Normal nutrition, no. (%)	At risk of malnutrition, no. (%)	p-value^1^	X^2^ (Cramer’s V)
AKI status				
AKI	30 (16%)	10 (5%)	0.733	0.12 (0.03)
Non-AKI	107 (57%)	41 (22%)		
Vital status				
Lived	96 (51%)	19 (10%)	<0.001*	16.82 (0.30)
Died	41 (22%)	32 (17%)		

Table 4 shows a statistically significant between-group difference for increased age (p=0.005) for patients at risk of malnutrition.

**Table 4:** Patient demographics contributing to risk of malnutrition ^1^Mann-Whitney U Test; *statistically significant <0.05; IQR: interquartile range

Demographics	Normal nutrition (n=137) median (IQR)	At risk of malnutrition (n=51) median (IQR)	p-value1	Z	U
Age (years)	82 (76-87)	86 (80-91)	0.005*	-2.824	2557.5
Height (m)	1.62 (1.55-1.70)	1.60 (1.53-1.65)	0.109	-1.603	2775.5
Weight (kg)	64.8 (57.1-75.9)	47.6 (44.2-56.7)	<0.001*	-7.049	1066.5
BMI (kg/m^2^)	24.7 (21.5-27.4)	19.2 (17.5-21.4)	<0.001*	-7.044	1048

Table 5 demonstrates a significant between-group difference based on nutrition status for serum biochemical markers, including preoperative and postoperative albumin and urea, as well as preoperative CRP and eGFR. Nutritional status had no significant effect on the electrolyte serum markers (i.e., sodium and potassium).

**Table 5:** Effect of nutrition on serum biochemical markers ^1^Mann-Whitney U Test; *statistically significant <0.05; CRP: C-reactive protein; eGFR: estimated glomerular filtration rate; Post-op: postoperative; Pre-op: preoperative

Test	Normal nutrition (n=137), median (IQR)	At risk of malnutrition (n=51), median (IQR)	p-value^1^	Z	U
Albumin (Pre-op) (g/L)	42.0 (40.0-45.0)	41.0 (38.0-43.0)	0.007*	-2.681	2607.5
Albumin (Post-op) (g/L)	35.0 (32.0-37.5)	34.0 (31.0-36.0)	0.018*	-2.375	2302.0
Creatinine (Pre-op) (umol/L)	72.0 (60.0-96.0)	85.0 (64.0-105.0)	0.272	-1.097	3129.5
Creatinine (Post-op) (umol/L)	73.0 (61.0-98.0)	77.5 (62.0-103.2)	0.755	-0.312	3274.0
CRP (Pre-op) (mg/L)	3.5 (1.1-12.3)	5.8 (1.8-35.1)	0.030*	-2.166	2776.0
CRP (Post-op) (mg/L)	127.7 (74.4 - 212.4)	128.6 (81.2 - 190.9)	0.918	-0.104	2047.5
eGFR (Pre-op) (ml/min/1.73m^2^)	71.0 (52.0-82.0)	55.0 (44.0-75.0)	0.039*	-2.066	2808.5
eGFR (Post-op) (ml/min/1.73m^2^)	72.0 (51.0-82.0)	66.5 (46.8-78.5)	0.337	-0.959	3065.0
Sodium (Pre-op) (mmol/L)	137.0 (134.5-139.0)	138.0 (133.0-140.0)	0.586	-0.544	3313.5
Sodium (Post-op) (mmol/L)	137.0 (134.0-139.0)	137.0 (133.8-140.2)	0.391	-0.858	3098.5
Potassium (Pre-op) (mmol/L)	4.1 (3.8 - 4.4)	4.3 (4.0 - 4.5)	0.194	-1.299	2388.0
Potassium (Post-op) (mmol/L)	4.1 (3.8 - 4.5)	4.1 (3.8 - 4.5)	0.720	-0.359	3235.0
Urea (Pre-op) (mmol/L)	6.6 (5.5-9.1)	7.3 (6.6-9.7)	0.013*	-2.472	2673.5
Urea (Post-op) (mmol/L)	6.1 (4.7-9.6)	7.5 (5.7-10.6)	0.030*	-2.175	2671.5

Table 6 shows a statistically significant between-group difference for delirium (p=0.001), as assessed by the 4AT score. A score of 0 indicates cognitive impairment/delirium is unlikely. A score of one to three indicates possible cognitive impairment. A score of four or more indicated possible delirium. In the context of our study, the 4AT data were collected from instances when it was performed when there was suspicion of delirium in patients during their inpatient stay. It also presents data to show statistically significant between-group differences for the total length of hospital stay (p=0.034), which can be used as a surrogate marker of a patient’s functional recovery back to a level fit for safe discharge from the hospital.

**Table 6:** Effect of extrinsic factors on risk of malnutrition ^1^Mann-Whitney U Test; *Statistically significant <0.05; 4AT: 4 A’s Test (Arousal, Attention, Abbreviated Mental Test - 4, Acute change); Post-op: postoperative

Variables	Normal nutrition (n=137), median (IQR)	At risk of malnutrition (n=51), median (IQR)	p-value^1^	Z	U
4AT score	0 (0-3)	3 (0-4)	0.001*	-3.288	2411.0
Post-op fluid balance (mls)	152.5 (-175.0-700.0)	564.5 (-168.8-1219.3)	0.391	-0.857	15.0
Time to surgery (hours)	21.8 (18.7-27.9)	25.8 (19.9-36.4)	0.091	-1.691	2828.0
Total length of hospital stay (days)	9.0 (6.0-12.5)	11.0 (7.0-17.0)	0.034*	-2.117	2793.0

## Discussion

The overall patient demographic of this study represents a predominantly elderly, female, and comorbid group of patients. This finding is in line with Lumbers et al. [8], where they concluded that a higher proportion of elderly female patients with hip fracture have malnutrition as compared to their age- and gender-matched cohort without a hip fracture. This is likely due to a combination of increased incidence of osteoporosis in females and the increased rate of falls in the elderly population generally [4, 8]. There have also been multiple studies [19-22] showing associations between nutrition and bone density, with malnourished patients being more prone to osteoporosis and, consequently, fractures. These studies have also highlighted the importance of appropriate nutrition, specifically protein, calcium, and vitamin D, in maintaining bone health and reducing age-related osteopenia and the associated risk of fractures.

Nutritional assessment performed on admission revealed that most patients (73%) had normal nutrition, and just over one-quarter (27%) were deemed at risk of malnutrition. The breakdown of the demographics revealed patients at risk of malnutrition to be older (p=0.005). A significant proportion of patients in this study were deemed at risk of malnutrition, but none of them reached the threshold of established malnutrition. Given that the nutritional assessment was done on admission, the implication is that this group was at risk prior to their hip fracture and hospital admission. Statistical analysis also showed a significantly increased risk of mortality (p<0.001) in patients at risk of malnutrition, with 63% of patients in this group who died, in contrast to only 30% in the group with normal nutrition, within 12 months of their hip fracture surgery. This demonstrates a twofold increase in mortality in the group of patients at risk of malnutrition. The findings of this study concur with existing literature reiterating the importance of optimizing nutrition for better patient outcomes [23-25].

This study also explored the association between nutrition and serum biochemical markers commonly requested as part of the baseline blood panel on admission. This study found a significant link between nutrition and albumin. It is the most abundant circulating protein found in plasma and can be susceptible to many factors influencing its levels, including inflammation and malnutrition, both of which can lower the concentration of albumin by decreasing the rate of synthesis [26]. This is particularly relevant in this specific cohort of patients with hip fractures, as it stands to reason that the albumin levels are likely to decrease after the fracture itself or the surgery to treat it, bearing in mind that they might also be low to begin with if they were malnourished prior to sustaining the fracture. Many studies have demonstrated that albumin has high sensitivity as a prognostic indicator for nutrition, with low levels being associated with longer length of hospital stay, higher rates of postoperative complications, and mortality [27,28]. As such, patients with hypoalbuminemia should be identified by the clinical team to ensure nutrition is optimized during the recovery period, and close monitoring of these patients should be undertaken, given the associated poor prognosis.

This study also found a small between-group difference for serum CRP, with patients at risk of malnutrition having slightly higher levels of CRP preoperatively but no significant difference postoperatively. Though more commonly used as a marker of inflammation or infection, CRP has been shown to have close links with nutrition, especially in acute disease. The Global Leadership Initiative on Malnutrition (GLIM), an organization whose purpose is to establish a global consensus on core diagnostic criteria for malnutrition, had proposed inflammation or disease burden as one of the etiologic criteria for the diagnosis of malnutrition [29]. Another study [30] built on this further by exploring the specific level of inflammation that correlates with low food intake in acutely ill hospitalized patients. They surmised that a serum CRP of 3.0 mg/dl and higher is a reasonable threshold of acute inflammation leading to reduced food intake. Merker et al. [29] investigated the association between baseline inflammatory status with nutritional support and showed a marked reduction in 30-day mortality in patients receiving nutritional support. This reduction, however, was not seen in patients classed as having high levels of inflammation (i.e., >10 mg/dl), implying the need for an individualized approach in planning for nutritional support. Though our study did not sub-classify the levels of CRP, the pre-operative findings are broadly in line with these studies, thereby validating CRP as an effective and readily available marker to serve as an adjunct in planning for nutritional support. However, CRP is known to rise substantially as a consequence of the physiological insult that accompanies major surgery (i.e., hip fracture surgery), and so its use in this context is limited postoperatively. The trauma of the hip fracture itself can also elevate the CRP, which should again be taken into consideration when interpreting preoperative results. 

In terms of renal biomarkers, this study found a significant association between malnutrition and both eGFR (pre-operatively) and urea (pre- and post-operatively), which is in line with other studies [31,32], but no significant association with creatinine. There is an inverse relationship between eGFR and malnutrition and a direct relationship between urea and malnutrition, as demonstrated by Guligowska et al. [32]. It is worth noting that the increase in urea could be a result of increased protein catabolism in states of malnourishment or dehydration, which could also account for the drop in renal function as a result of AKI. As such, further interventional studies on the effects of nutritional therapy on these specific biomarkers are required to prove causality between nutrition and renal function.

Lastly, this study demonstrated a significant association between nutrition and the development of delirium as well as an increased length of total hospital stay. Delirium is a transient acute neurocognitive disorder that is common, especially amongst elderly inpatients, in which malnutrition is one of the commonly recognized precipitants [33,34]. Not only does it complicate the recovery of patients from surgery, leading to higher rates of mortality, but it is also a significant factor for healthcare staff and family members looking after the patient, and increases the cost burden to the national healthcare service [35].

Overall, this study concurs with existing literature in that nutrition plays an integral role in determining the clinical outcomes of patients with hip fractures. It has highlighted certain demographic characteristics most at risk, namely the elderly, female, and comorbid, which is important in the context of public health and preventative medicine. The findings of this study have also shown the detrimental clinical outcomes associated with patients at risk of malnutrition, including increased mortality rate, development of delirium, and longer hospital stays. Finally, it has demonstrated certain laboratory markers that were significantly linked to nutrition status, including albumin and urea, and suggests that these be incorporated as part of early nutritional assessment.

Early intervention, such as activating food charts and referring to dieticians or nutrition support teams for advice on nutritional supplementation and food choices to accommodate special diets, in addition to identification of the cause of malnutrition, is important. Continued assessment following identification is also equally important to ensure correction and maintenance of good nutrition. This can be in the form of repeated screenings weekly whilst in hospital or monthly in care homes or during scheduled visits in the community by the community team post discharge, at the discretion of local hospital policies.

Future interventional research into the effects of nutritional therapy on specific biochemical markers (e.g., albumin, urea, CRP) in relation to their use as nutrition and prognostic markers would be helpful in aiding clinicians in tailoring care to optimize overall hip fracture care. Further prospective research can also be conducted to evaluate the recurrence rate of fractures in patients identified to be at risk of malnutrition who have received nutrition optimization therapy. Further investigation of cut-off thresholds for key serum biomarkers would also be valuable in refining clinical practice.

A key strength of this study is the broad range of clinical outcomes assessed, including mortality, delirium, and length of stay, which allows for a comprehensive comparison between the two groups. However, several important limitations must be acknowledged. The study was conducted at a single center and employed a retrospective design, which may limit generalizability and introduce selection bias. Additionally, data on perioperative complications and functional recovery-such as activities of daily living (ADL) at discharge and rates of discharge to home versus care facilities-were not collected. Inclusion of such measures would have strengthened the clinical impact by offering a more holistic understanding of patient recovery and long-term outcomes. The study also lacked longitudinal nutritional assessments, which may have provided insights into how changes in nutritional status over time influence outcomes. Future research should aim to incorporate these elements and utilize multivariate logistic regression and receiver operating characteristic (ROC) analyses to identify and validate significant predictors of poor outcomes.

## Conclusions

Hip fractures remain a common and significant cause of hospital admissions, particularly among elderly, comorbid females. This study highlights the critical role of nutritional status in influencing clinical outcomes in hip fracture patients. The findings indicate that a notable proportion of patients were deemed at risk of malnutrition, which was strongly associated with increased mortality, higher rates of delirium, and longer hospital stays. Serum biochemical markers, including low albumin and elevated urea, were significantly associated with malnutrition, suggesting their potential utility as adjunctive indicators for early nutritional risk stratification. However, the study's single-center, retrospective design, lack of longitudinal nutritional assessment, and absence of functional outcome measures such as activities of daily living or discharge destination may limit the generalizability and scope of the findings. Despite these limitations, the results support the integration of nutritional assessment and management as a core component of hip fracture care to enhance patient outcomes, reduce hospital stay durations, and alleviate socioeconomic healthcare burdens.
